# Integrating Phenological Features with Time Series Transformer for Accurate Rice Field Mapping in Fragmented and Cloud-Prone Areas

**DOI:** 10.3390/s25247488

**Published:** 2025-12-09

**Authors:** Tiantian Xu, Peng Cai, Hangan Wei, Huili He, Hao Wang

**Affiliations:** 1 Jiangsu Key Laboratory of Atmospheric Environment Monitoring and Pollution Control (AEMPC), Collaborative Innovation Center of Atmospheric Environment and Equipment Technology (AEET), Joint International Research Laboratory of Climate and Environment Change (ILCEC), School of Environmental Science and Engineering, Nanjing University of Information Science & Technology (NUIST), Nanjing 210044, China; tt.x@nuist.edu.cn (T.X.);; 2 Key Laboratory of Ecosystem Carbon Source and Sink, China Meteorological Administration (ECSS-CMA), Wuxi University, Wuxi 214105, China; 3 Jiangsu Key Laboratory for Recognition and Remediation of Emerging Pollutants in Taihu Basin, School of Atmospheric Science and Remote Sensing, Wuxi University, Wuxi 214105, China

**Keywords:** rice identification, time series analysis, satellite image, SITS-Former, phenological features

## Abstract

Accurate identification and monitoring of rice cultivation areas are essential for food security and sustainable agricultural development. However, regions with frequent cloud cover, high rainfall, and fragmented fields often face challenges due to the absence of temporal features caused by cloud and rain interference, as well as spectral confusion from scattered plots, which hampers rice recognition accuracy. To address these issues, this study employs a Satellite Image Time Series Transformer (SITS-Former) model, enhanced with the integration of diverse phenological features to improve rice phenology representation and enable precise rice identification. The methodology constructs a rice phenological feature set that combines temporal, spatial, and spectral information. Through its self-attention mechanism, the model effectively captures growth dynamics, while multi-scale convolutional modules help suppress interference from non-rice land covers. The study utilized Sentinel-2 satellite data to analyze rice distribution in Wuxi City. The results demonstrated an overall classification accuracy of 0.967, with the estimated planting area matching 91.74% of official statistics. Compared to traditional rice distribution analysis methods, such as Random Forest, this approach outperforms in both accuracy and detailed presentation. It effectively addresses the challenge of identifying fragmented rice fields in regions with persistent cloud cover and heavy rainfall, providing accurate mapping of cultivated areas in difficult climatic conditions while offering valuable baseline data for yield assessments.

## 1. Introduction

Rice, as one of the world’s most vital staple crops, serves as the primary food source for over half the global population [1]. The extent of its cultivation area, along with its growth status and yield, has a direct impact on national food security [2], water resource management [3], and ecological conservation [4]. With the ongoing growth of the global population and the intensification of climate change, the precise monitoring and management of cultivation areas and growth conditions for key crops such as rice have become critical tasks for agricultural research and national food security [5].

As one of the world’s largest rice producers, China has an annual output of about 206 million tons, accounting for roughly 28% of the global total [6]. China’s rice cultivation area, output, and economic value form the cornerstone of national food security and help stabilize the agricultural economy and global food markets [7]. This importance is especially strong in the eastern coastal regions, which act as the country’s main grain hinterland. Rice farming is highly concentrated there. Wuxi City, a typical representative of this area, sits on the Jiangsu-Zhejiang Plain. The plain is a broad, flat alluvial area formed by river networks [8]. Its soils are diverse, mainly fertile loam and clay loam, which support productive rice farming and help establish Wuxi as a major production base [9]. However, frequent cloudy and rainy weather and highly fragmented farmland make precise monitoring of rice cultivation challenging. This fragmentation has been intensified by urban expansion and land-use transitions, during which agricultural land is continually subdivided and encroached upon, resulting in scattered field distribution and generally small plot sizes. Studies based on parcel-scale evaluation of commercial land-use intensification have highlighted the pronounced conflict between construction land growth and farmland conservation under rapid urbanization in Wuxi, where commercial and agricultural uses are spatially interwoven, further aggravating the discontinuity and dispersion of farmland [10]. Concurrently, research on urban development boundary demarcation reflects an increasingly complex land-use pattern in Wuxi, with mixed functions in peri-urban areas, erosion of agricultural space, and field structures fragmented by transportation, residential, and industrial uses, forming a mosaic-like distribution [11]. Such a fragmented land-use pattern not only diminishes agricultural economies of scale but also poses considerable difficulties for field management, crop monitoring, and yield estimation. This situation calls for more advanced technological solutions to ensure accurate monitoring and resilience in the sector.

With the development of remote sensing technology, optical multispectral imagery has seen increasingly widespread application in agricultural monitoring [12]. Current rice identification methods primarily fall into three categories: traditional approaches based on vegetation index thresholds [13,14], machine learning methods [15,16], and deep learning methods [17,18].

Vegetation index-based rice identification typically relies on fixed or dynamic thresholds to distinguish rice from other land cover types [19]. However, factors such as saturation effects, weed interference, and soil background variations often lead to poor recognition performance in practical applications, particularly in fragmented or small-scale rice cultivation areas [20]. Moreover, these methods exhibit low sensitivity to vegetation indices, making it challenging to fully capture the complex developmental changes across different rice growth stages [21]. In contrast, machine learning methods like Random Forest (RF) and Support Vector Machine (SVM) can integrate multispectral and multi-feature information, improving classification performance to some extent [22,23]. However, these approaches usually treat features from different time points as independent data, overlooking the inherent dynamic temporal patterns of rice growth. This limitation hinders machine learning models from fully capturing complex spatiotemporal relationships, leading to reduced recognition accuracy, especially in cloudy areas [24].

With the rise of deep learning, methods like Convolutional Neural Networks (CNN) and Recurrent Neural Networks (RNN) have demonstrated superior performance [25,26]. These models autonomously learn complex spatio-temporal features, significantly improving rice recognition accuracy. However, existing deep learning models predominantly focus on either spatial feature extraction or time series analysis, failing to effectively integrate both advantages [27,28]. Long Short-Term Memory (LSTM) networks, as a specialized type of RNN, leverage their robust time series modeling capabilities to effectively capture rice growth patterns while exhibiting resilience to data gaps and noise [29,30]. These methods not only struggle with limitations in handling extremely long sequences and integrating spatial information, but also struggle against spatial heterogeneity challenges arising from fragmented farmland, failing to efficiently capture long-range dependencies across entire growing seasons [31]. Furthermore, errors tend to accumulate over time under continuous cloud and rain interference.

Recent advances in deep learning have driven innovations in remote sensing, enabling the more accurate capture of complex spatiotemporal features. In temporal sequence modeling, models such as TempCNN and TCN address the limitations of earlier approaches, outperforming traditional recurrent networks such as LSTM in certain scenarios [32,33]. However, most methods still emphasize either spatial or temporal aspects and struggle to integrate both. Challenges remain in handling spatial heterogeneity in fragmented farmlands, modeling long-term dependencies across entire growing seasons, and mitigating error accumulation caused by clouds and rain. These issues underscore the need for more robust temporal modeling solutions.

To overcome these limitations, researchers have introduced transformer architectures into remote sensing time series analyses [34,35,36]. Within this framework, SITS-Former leverages its core self-attention mechanism to process entire sequences in parallel, effectively capturing long-term dependencies and flexibly integrating multisource information, marking a significant advancement in land cover classification [37]. However, this model still faces considerable challenges in the monitoring of specific crops. Existing methods struggle to fully capture the long-term temporal dependencies throughout the entire rice growth cycle, making it difficult to track complete phenological trajectories [38,39]. However, partial data loss in areas with high cloud cover and heavy precipitation further impedes the reconstruction of complete growth patterns [40,41,42].

To address the challenges of rice identification in regions characterized by frequent cloud cover, intense rainfall, and fragmented fields, this study developed a monitoring framework based on Sentinel-2 time series imagery. The core innovation involves enhancing the SITS-Former model to broaden its applicability to complex agricultural environments. Existing applications of SITS-Former primarily focus on general land cover classification, often utilizing simple feature embeddings and fixed positional encodings. We propose targeted improvements for the precise monitoring of rice crops. The specific contributions of this study are as follows: (1) Constructing a rice phenological feature set that integrates temporal, spatial, and spectral information to compensate for the scarcity of clear-sky observations in cloudy regions; (2) Introducing the time series Difference Vegetation Index (TDVI) and Normalized Change Rate Vegetation Index (NCRVI) to improve the detection of dynamic changes during critical growth stages; (3) Designing a parallel three-branch embedding structure combined with channel attention to enhance feature discriminability; proposing a seasonal pattern-day-age projection encoding to capture phenological stages; and innovatively introducing a phenological attention gating module that enables transformers to dynamically amplify key growth signals based on phenological context; (4) Validating the framework in cloud-heavy, rainy, and fragmented farmland environments such as Wuxi, demonstrating that it achieves high-precision rice distribution mapping by capturing long-range dependencies through self-attention and suppressing spatial interference via multi-scale convolutions. This provides a reliable technical solution for monitoring the fragmentation of agricultural areas.

## 2. Study Area and Materials

### 2.1. Study Area

**Figure 1:**
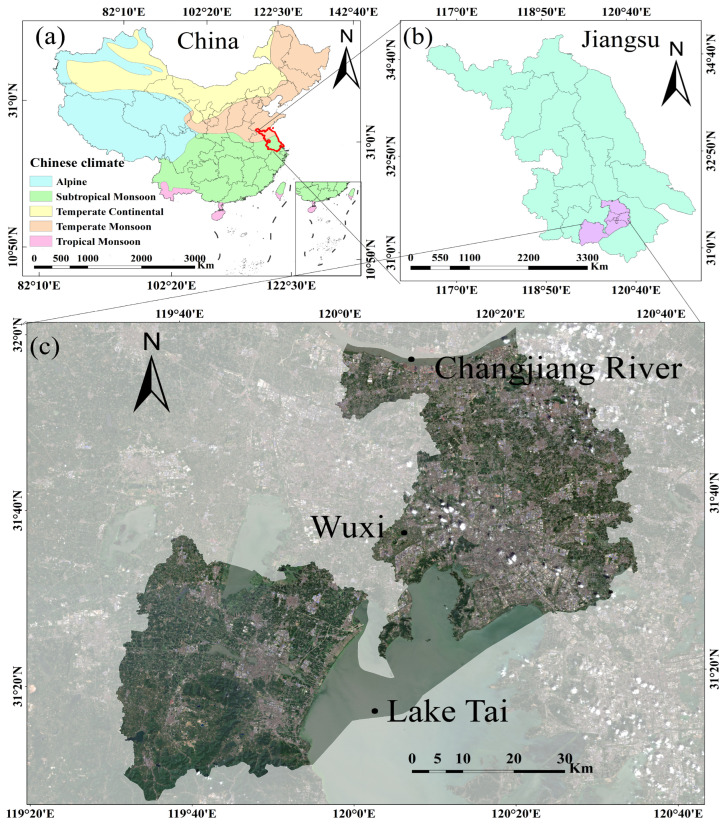
Geographical Location and Climatic Background of the Study Area: (**a**) Climate distribution map of China, with Jiangsu Province highlighted in red; (**b**) administrative map of Jiangsu Province, China, with Wuxi’s geographical location highlighted in purple; (**c**) September Sentinel-2 image of the Wuxi study area.

Wuxi City, situated in southern Jiangsu Province in the heart of China’s eastern Yangtze River Delta, serves as the geographical context for this study. Its precise location is defined by coordinates of 31°7′ to 32°2′ north latitude and 119°33′ to 120°18′ east longitude (Figure 1). The study area experiences a northern subtropical monsoon climate, which is distinguished by pronounced seasonal shifts. Notably, summers (June–August) are characterized by warm temperatures, abundant rainfall, and pervasive cloud cover. This prevalent cloudiness and precipitation during the summer months, which coincides with the critical rice-growing season, presents a significant impediment to the consistent acquisition of high-quality optical remote sensing data. In contrast, winters are typically dry and clear. Wuxi experiences an average annual temperature of 16.2 °C and receives approximately 1000 mm of rainfall annually [43]. Geographically, the city is bounded by the Yangtze River to the north and expansive Lake Tai to the south. These favorable climatic and hydrological conditions are conducive to rice cultivation. However, rapid urban development has led to the fragmentation of traditional rice-growing areas, creating a landscape of dispersed agricultural plots. To quantitatively assess this spatial pattern, we integrated field survey data, visual interpretation of high-resolution remote sensing imagery, and statistical data to derive the mean paddy field patch area within each administrative district. The results reveal a distinct fragmentation gradient: Xinwu and Binhu Districts exhibit significantly smaller mean patch areas, reflecting a high degree of farmland fragmentation driven by urban expansion.

**Figure 2:**
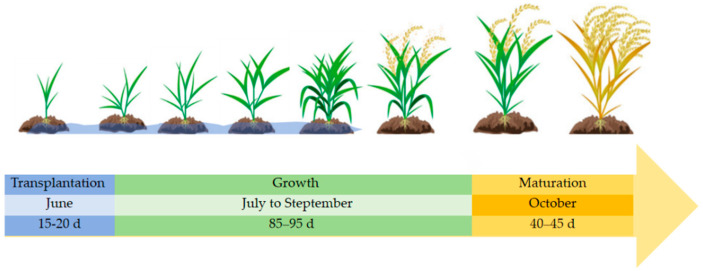
Diagram of rice growth process in Wuxi.

Wuxi City is located in the eastern plains of China and is one of the primary rice-growing regions in the country. In the study area (Wuxi City), rice cultivation primarily follows a single-season planting pattern, characterized by a typical phenological cycle that can be divided into transplanting, growth, and maturation stages (Figure 2). The region predominantly cultivates single-season japonica rice varieties, which exhibit relatively similar phenological cycles. This standardized cultivation practice helps reduce intra-regional variability caused by differences in rice varieties or management practices.

To provide a more precise understanding of these stages within the local context, we delineated the duration of each phenological period. Specifically, the transplanting period, during which seedlings are moved to the field and enter the tillering recovery phase, lasts approximately 15–20 days. The subsequent growth period, spanning from late June to early September, encompasses approximately 85–95 days. This crucial phase encompasses key developmental stages, including tillering, stem elongation, and heading, and represents the core period for rice biomass accumulation. Finally, the ripening period, from mid-September to late October, lasts approximately 40–45 days, during which rice progresses from grain filling to seed maturity, culminating in harvest.

### 2.2. Data

This study utilizes Sentinel-2 satellite L2A-level surface reflectance products (MSI). The satellite’s multispectral imager (MSI) has 13 spectral bands. Specific details are shown in Table 1. All imagery underwent radiometric calibration and atmospheric correction (L2A) preprocessing. Data captured by the MSI sensor spans the full spectral range of visible light, near-infrared, and shortwave infrared. The red edge bands (comprising one near-infrared and two shortwave infrared bands) effectively reflect crop growth conditions, making them suitable for crop classification, growth assessment, and yield prediction [44]. All Sentinel-2 data were accessed via the Copernicus Data Center (https://dataspace.copernicus.eu/, accessed on 15 March 2025).

This study selected a cloud cover threshold of 30% for the single-image screening. Considering that July and August in Wuxi coincide with the rainy and typhoon seasons, resulting in frequent cloud cover, setting the threshold too low could lead to insufficient usable images, thereby affecting subsequent time-series analysis. Therefore, the 30% threshold ensures image quality while retaining sufficient time-series data to meet the requirements of phenological analysis.

The study period from June to October was chosen because it closely aligned with the complete growth cycle of single-season rice in Wuxi. Early to mid-June marks the rice transplanting period, July to September represents the critical phase for both vegetative and reproductive growth, and late October signals the harvest season. This cycle comprehensively captures all phenological stages from transplantation to harvest, along with the corresponding spectral feature changes. The specific data are presented in Table 2.

## 3. Methodology

**Figure 3:**
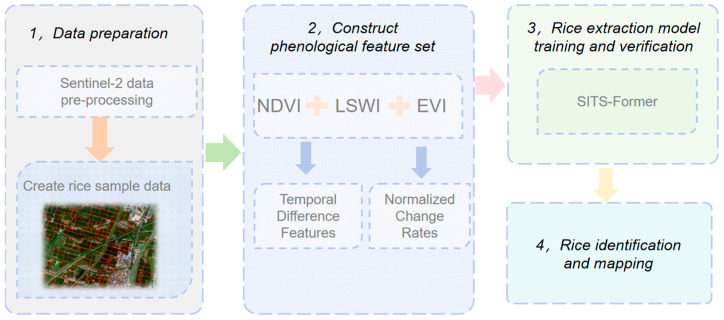
Research method flow chart.

The research workflow comprises data preprocessing, construction of the rice sample dataset, establishment of phenological feature sets, training and validation of the rice extraction model, and rice identification (Figure 3).

### 3.1. Data Preparation

To address data gaps in Sentinel-2 L2A products caused by frequent cloud cover and rainfall during the summer in Wuxi, this study employed advanced cloud removal and time-series reconstruction techniques to generate continuous, high-quality rice growth trajectories. Specifically, the Sentinel-2 L2A image collection was loaded into Google Earth Engine (GEE, Google LLC, Mountain View, CA, USA), and the built-in Scene Classification Layer (SCL) was used to mask clouds, cloud shadows, snow, and water at the pixel level. Pixels identified as cloud shadows, medium- and high-probability clouds, and cirrus were excluded to minimize interference.

To compensate for the data gaps caused by cloud cover, this study employed time-series reconstruction and smoothing techniques. First, vegetation index time series, including NDVI, LSWI, and EVI, were constructed for each pixel using Sentinel-2 L2A data, and preliminary noise reduction was applied using the Savitzky-Golay filtering. Subsequently, a Harmonic Regression model was employed to capture periodic vegetation growth patterns and accurately estimate vegetation indices during cloud-obscured periods (represented as NaN values). This process successfully reconstructed smooth, continuous trajectories of the vegetation index throughout the rice-growing season (June-October), enabling precise detection of key phenological transitions. The resulting gap-filled and smoothed time series provided a reliable basis for subsequent calculations of TDVI and NCRVI, significantly enhancing the accuracy and representativeness of the derived features as inputs to the SITS-Former model.

To address the reviewer’s concern regarding the impact of cloud cover, we acknowledge that frequent clouds and rain in Wuxi significantly reduced the number of clear-sky observations. Although this study does not provide a quantitative analysis of the exact percentage of temporal data lost to clouds at each pixel, our methodology is specifically designed to mitigate this challenge. The extensive use of Google Earth Engine (GEE, Google LLC, Mountain View, CA, USA) for data acquisition and the subsequent application of advanced time series reconstruction techniques, including Harmonic Regression, are crucial for filling these data gaps. This approach ensured we obtained continuous, smoothed vegetation index trajectories, effectively compensating for missing clear-sky observations and enabling the SITS-Former model to learn robust phenological patterns despite intermittent data availability.

To ensure the spatial consistency of the imagery and maintain the accuracy of subsequent index computations and spatial analyses, all Sentinel-2 L2A products were resampled. This procedure was performed using the SNAP 9.0 software (European Space Agency, Frascati, Italy). First, all 13 MSI bands were imported in a batch. Then, the “Resample” tool was applied with the target spatial resolution set to 10 m and the bilinear interpolation method selected, while retaining the default parameters. Finally, batch resampling was conducted on all the images during the study period. This step ensured the spatial alignment of pixels across the entire dataset, establishing a consistent and stable spatial baseline for constructing temporal features and providing input to the SITS-Former model.

### 3.2. Constructing the Phenological Feature Set

Rice growth follows distinct phenological cycles; therefore, extracting phenological features from time-series remote sensing data is important for accurate identification. This study selected the Normalized Difference Vegetation Index (NDVI), Land Surface Water Index (LSWI), and Enhanced Vegetation Index (EVI) to form a phenological feature set [45]. Among these, NDVI effectively reflects vegetation coverage and growth status, LSWI is sensitive to canopy moisture content and rice field flooding conditions, and EVI is more suitable for monitoring during periods of high biomass by reducing interference from atmospheric and soil background factors [25,46]. In addition, the Time Difference Vegetation Index (TDVI) and Normalized Change Rate Vegetation Index (NCRVI) were included to improve the description of rice phenology, as these indices complement each other across dimensions such as vegetation greenness, moisture response, and change dynamics. Compared to single indices or other alternative indicators, they provide a more comprehensive description of the unique phenological trajectory of rice [47,48].

NDVI is highly sensitive to leaf area index and leaf cover, directly reflecting greenness, and is often used as a key indicator of rice biomass. During rice identification, monitoring changes in vegetation cover across growth stages helps distinguish rice from other crops [49]. After transplanting, NDVI rises quickly, peaks at tillering, and then gradually declines during grain filling. This pattern contrasts with what is observed in dryland crops. NDVI is typically calculated by comparing signals from the near-infrared and red bands, with the formula expressed as:

(1) $$NDVI=\frac{B8-B4}{B8+B4}$$

where B8 denotes reflectance in the Sentinel-2 near-infrared band (NIR_1), and B4 denotes reflectance in the Sentinel-2 red band (Red).

LSWI mainly shows the condition of water on the surface. It is very sensitive to water stress and how well rice plants are able to use water. This helps tell rice apart from crops grown on dry land. During the flooded cultivation phase, fields usually have a layer of water, which leads to generally higher LSWI values [50]. Upland crops, however, have lower LSWI values because they have less soil moisture. Also, LSWI drops considerably when rice enters the drying stage. This change in moisture is a key sign for identifying rice, especially in southern areas that use multiple cropping systems. Its formula is:

(2) $$LSWI=\frac{B8-B11}{B8+B11}$$

where B11 denotes reflectance in the Sentinel-2 short-wave infrared band (SWIR_1).

EVI includes blue light in its calculations and effectively reduces soil background noise. This lets it more accurately reflect crop growth changes, especially in areas with dense vegetation [51]. This feature gives it significant advantages when monitoring lush crops. The EVI formula is as follows:

(3) $$EVI=2.5\frac{B8-B4}{7B8-6B2+1}$$

where B2 denotes reflectance in the Sentinel-2 blue band (Blue).

#### 3.2.1. Temporal Difference Vegetation Index

The TDVI reflects changes in rice growth by analyzing shifts in vegetation indices between two consecutive time periods (*t* and *t* + Δ*t*) [52]. Different growth stages of rice have a significant impact on vegetation index measurements. During the tillering stage, rice produces many new shoots, which increases the number of leaves and chlorophyll content and leads to a large rise in NDVI. At this stage, TDVI values are positive, indicating the plant is in a rapid growth phase. When entering the ripening stage, crop leaves start to age and gradually turn yellow. As chlorophyll content decreases and plant activity drops, vegetation indices fall quickly. Negative TDVI values clearly reflect the rate of plant senescence. By tracking TDVI’s shifts between positive and negative values, key growth stages of rice can be accurately identified. Its calculation formula is:

(4) $$TDVI=VI_{t+\Delta t}-{VI}_{t}$$

where *VI* denotes the vegetation index (NDVI, LSWI, or EVI), *t* represents time, and Δ*t* is the time interval between adjacent rice growth nodes.

#### 3.2.2. Normalized Change Rate Vegetation Index

NCRVI represents the relative change in vegetation indices over a given period. It reduces baseline effects, enabling comparisons across time and regions. Even with the same rice variety, differences in soil fertility, irrigation, and fertilizer use can cause large differences in the absolute values of vegetation indices like NDVI across plots. For example, NDVI is usually higher in fertile fields than in infertile ones. By normalizing the index, NCRVI removes these baseline differences, making the relative growth rates between two fields more comparable within the same period [53].

NCRVI can also reveal growth stagnation or decline. For instance, during the harvest season, consistently negative NCRVI values often indicate rapid senescence in rice. If NCRVI falls well below historical or average levels during the active growth period, it may suggest growth constraints such as nutrient deficiency, disease, or water stress. Its calculation formula is:

(5) $$NCRVI=\frac{VI_{t_{2}}{-VI}_{t_{1}}}{{VI}_{t_{1}}}\times 100\%$$

where *t*_1_ is the start time and *t*_2_ is the end time.

### 3.3. SITS-Former Model

**Figure 4:**
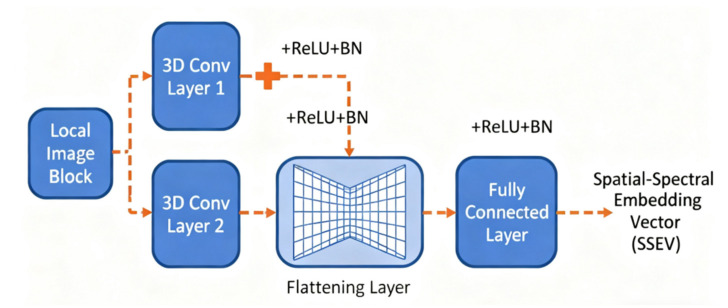
Illustration of the spatio-spectral embedding network.

**Figure 5:**
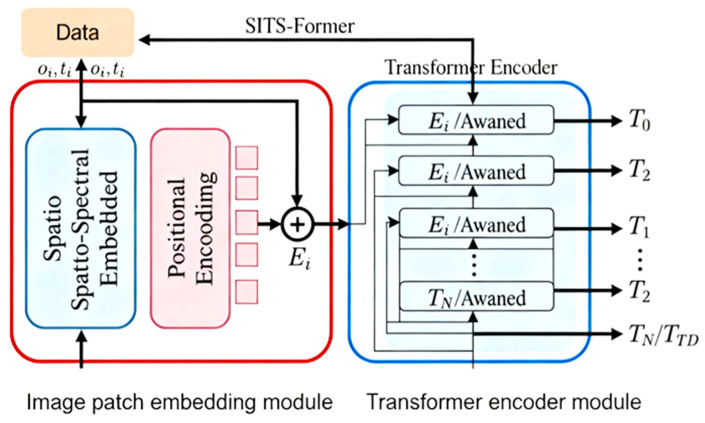
SITS-Former model architecture diagram.

This method achieves accurate rice identification by combining the SITS-Former model with a newly developed feature set construction approach. The SITS-Former model has two primary components: a pixel-level embedding module and a Transformer encoder module [54,55]. These modules extract deep spatial, spectral, and temporal information from remote sensing time series data. The image block embedding module analyzes each local image block to create a spatial-spectral embedding vector (SSEV). This is accomplished through a lightweight network comprising two 3D convolutional layers, a flattening layer, and a fully connected layer (Figure 4). Temporal data is captured in a position embedding vector (PEV), generated using sine position encoding. These two vectors are then merged into a single, unified input embedding vector Ei. The Transformer encoder module processes these embedded vectors sequentially, detecting temporal relationships through multiple attention and feed-forward layers. The multi-head attention mechanism transforms input sequences into Q, K, and V matrices using separate fully connected layers. Attention weights, which measure time-based relationships between data points, are calculated through dot product operations. This process yields detailed time-based features for subsequent operations. The system’s design is illustrated in Figure 5.

The SITS-Former model leverages self-attention to identify both changes and relationships within vegetation index time series data. This capability effectively addresses challenges related to long-term dependencies in time series information [23,56]. The model achieves superior classification results by integrating features from different time points. It offers an optimal solution for analyzing multi-dimensional features and time-based patterns in remote sensing data. The SITS-Former model utilizes all available temporal vegetation index data from Sentinel-2 satellites, processing core metrics such as NDVI, LSWI, and EVI [57]. The model precisely tracks changes in vegetation indices throughout the various stages of rice development.

The self-attention mechanism within this model determines which vegetation indices are most significant at each temporal point [58]. This allows the model to focus on crucial rice growth phases and their corresponding index patterns, leading to precise rice growth monitoring capabilities. The model effectively handles Wuxi’s complex land use patterns by utilizing its robust temporal data processing abilities. This resolves analytical issues that arise when rice and other vegetation exhibit similar spectral patterns at specific times. SITS-Former employs time-based growth patterns to detect rice fields across different land sections, resulting in enhanced detection performance.

While building upon the established SITS-Former framework, this study introduces key architectural innovations to enhance phenological sensitivity in crop identification. Rather than merely adopting a generic 3D-Conv + Transformer hybrid, we propose two synergistic enhancements: a novel Phenology Gate mechanism that serves as an attention variant, dynamically modulating temporal attention based on phenological context, and an adaptive multi-branch embedding structure with channel-wise attention that refines feature fusion. These components work together to improve discrimination of crops with similar spectral signatures but distinct growth patterns, particularly under fragmented and cloud-prone conditions.

Specifically, we redesign the input embedding using a parallel three-branch architecture that captures direct features, deep abstractions, and feature interactions. These multi-scale representations are fused and recalibrated via channel attention, enhancing input discriminability. Building on this, two Conv3D layers are added for temporal feature extraction, with a stride of 1, padding = ‘same’, and 64 filters; dropout is set to 0.1. When calculating features, NDVI, LSWI, and EVI are computed at each valid time step, while TDVI and NCRVI are computed over consecutive valid time steps. For temporal encoding, we introduce adaptive positional embeddings—Seasonal Pattern Encoding and DOY Projection Encoding—to incorporate phenological context into the Transformer. Within the encoder, the Phenology Gate module, implemented as a lightweight MLP, generates dynamic gating weights that scale transformer outputs, sharpening focus on critical growth stages. The encoder consists of 4 layers with 8 attention heads, a model dimension of 64, and a feed-forward dimension of 256, with dropout set to 0.1 throughout.

Finally, temporal features are aggregated using global average pooling. These aggregated representations are processed by a two-layer MLP classifier and a Softmax output layer, enabling precise, interpretable identification of rice growth stages even under challenging real-world conditions.

### 3.4. Experimental Setup

#### 3.4.1. Training Sample Generation

This study used 10 m-resolution Sentinel-2 satellite imagery as its primary data source. By combining field surveys with high-resolution visual interpretation of the imagery, it obtained highly reliable ground-truth data. The study combined high-resolution imagery and field surveys to manually delineate 5000 rice polygon areas and 5000 non-rice areas through visual interpretation. A cross-verification mechanism involving manually drawn polygons ensured rigorous accuracy in both boundary delineation and land-use classification. The sample data covered diverse land cover types, including urban structures, forested areas, water bodies, and other crops, comprehensively reflecting the surface vegetation characteristics of the study area.

To ensure sufficient training data and reliable evaluation results, a stratified sampling strategy was used to generate the sample. During polygon construction, particular emphasis was placed on controlling the area proportions of each land cover type to precisely match the actual land cover distribution pattern of the study area. To further expand the sample size and enhance information richness, ArcGIS 10.8 software (Esri Inc., Redlands, CA, USA) was used to randomly generate 30,000 sampling points within the polygonal areas. To reduce spatial autocorrelation and ensure spatial independence of samples, a minimum spacing constraint of 10 m was applied to the sampling points. Finally, the samples were annotated into a binary category based on land type attributes: paddy fields were labeled as 1, and non-paddy areas as 0.

Data partitioning employs a strict spatial isolation strategy to ensure model generalization and prevent overestimation of performance. While the initial description mentions using scikit-learn’s train_test_split function, this is performed at the polygon level rather than on individual points. Specifically, each of the initial 10,000 polygons was assigned a unique identifier. All sampled points generated from the same polygon inherited this identifier. We then employed scikit-learn’s GroupShuffleSplit or StratifiedGroupKFold functions, passing the polygon identifiers as the groups parameter. This approach ensures that all sample points from the same polygon are assigned exclusively to either the training or test set, effectively severing spatial dependencies between the training and test sets. This guarantees the test set’s spatial independence, providing a more realistic and rigorous assessment of the model’s performance in unseen areas. The final dataset comprises 24,000 training samples and 6000 test samples. Feature standardization was performed using StandardScaler, where scaling parameters (mean and standard deviation) derived solely from the training set were applied to both subsets to prevent data leakage.

#### 3.4.2. Training Strategies

To enhance SITS-Former’s temporal feature characterization and mitigate overfitting, this study optimized the network architecture. Xavier uniform distribution initialized the input projection layer weights, with biases set to zero for training stability. The feedforward network dimension of the Transformer encoder layer was reduced from 4× to 2× hidden dimension to simplify complexity and prevent overfitting. The ReLU activation function was replaced with GELU to increase performance stability. To better utilize information from all time steps, global average pooling was adopted instead of using only the last time step output.

The model uses a temporal processing architecture with input projection layers, positional encoding, multi-head attention mechanisms, a Transformer encoder, and a classification head. Training used AdamW optimizer with 0.001 learning rate and cosine annealing scheduling. Cross-entropy loss was used, with training over 100 epochs using a 32-batch size. Global gradients underwent norm clipping with a 1.0 threshold. ReduceLROnPlateau served as a learning scheduler. These parameters were optimized through initial validation to balance convergence speed and generalization performance.

All experiments in this paper were conducted in PyCharm Community Edition 2024.2.4 (JetBrains s.r.o., Prague, Czech Republic), using Python 3.9, with an NVIDIA GeForce RTX 3090 GPU (NVIDIA Corporation, Santa Clara, CA, USA).

#### 3.4.3. Validation Indicators

To validate the accuracy of rice mapping results, we utilized the “Wuxi Statistical Yearbook (2024)” as the data source. For algorithm evaluation, we extracted Wuxi’s cultivated area data for comparative analysis. In this comparison, statistical rice distribution data served as actual data, while our mapping results acted as predicted data. Subsequently, we employed a confusion matrix as a validation tool, comparing field truth samples with selected expanded samples. Evaluation metrics included accuracy, precision, recall, F1 score, and the kappa coefficient. Detailed explanations of these parameters are provided in Table 3.

In Table 3, *TP* denotes the number of rice samples correctly identified. *TN* represents the number of non-rice samples correctly identified. *FP* indicates the number of non-rice samples incorrectly classified as rice. *FN* signifies the number of rice samples incorrectly classified as non-rice. *Po* is the observed consistency (i.e., Accuracy), while *Pe* is the expected value of random consistency.

## 4. Results

### 4.1. Model Performance Evaluation

**Figure 6:**
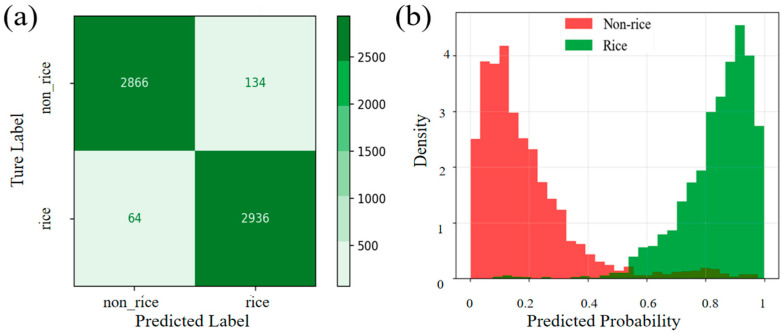
(**a**) Confusion Matrix; (**b**) Probability density prediction map.

This study evaluated the performance of the proposed SITS-Former model in rice and non-rice classification using confusion matrices and prediction probability density plots (Figure 6). As shown in Figure 6a, the model correctly identified 2866 non-rice samples and 2936 rice samples, with both categories demonstrating low false-positive rates. Figure 6b shows that non-rice samples were predominantly predicted in the low probability range, whereas rice samples were concentrated in the high range. The nearly non-overlapping probability distributions between these two groups demonstrate clear confidence distinctions in the classification, further validating the classification capabilities of the model.

### 4.2. Model Performance Comparison

To rigorously evaluate the SITS-Former model’s capabilities in rice field detection and mapping, we designed a comprehensive benchmarking framework. Considering the complexity of current methods in rice remote sensing mapping, we constructed a series of extensive benchmarks, including traditional machine learning approaches (Random Forest RF), foundational deep learning models (CNN, LSTM), and representative advanced models in current remote sensing time series analysis, such as TempCNN, Temporal Convolutional Networks (TCN), and foundational Transformer architectures.

All comparison models underwent identical data preprocessing workflows, including consistent data cleaning strategies (handling missing and outlier values), vegetation index calculations (NDVI, EVI, LSWI, and our proposed TDVI and NCRVI), data normalization methods, and strict training/test set partitioning. This consistent design ensures fair model performance comparisons while highlighting differences in feature extraction capabilities under identical input conditions.

In terms of implementation, each comparative model was constructed and optimized based on its typical architecture. The Random Forest (RF) model utilized scikit-learn’s RandomForestClassifier with n_estimators = 500, max_depth = None, min_samples_split = 2, and min_samples_leaf = 1. The Convolutional Neural Network (CNN) was implemented using TensorFlow/Keras, consisting of three convolutional layers with 32, 64, and 128 filters, each having a 3 × 3 kernel size, followed by max-pooling (2 × 2) and ReLU activation. The Long Short-Term Memory (LSTM) model adopted a two-layer structure with 128 hidden units per layer, followed by a fully connected layer for classification. The Temporal Convolutional Neural Network (TempCNN) employed three 1D convolutional layers with 32, 64, and 128 filters and a kernel size of 3, each followed by batch normalization and ReLU activation, with classification performed via global average pooling and a fully connected layer. The Temporal Convolutional Network (TCN) incorporated four residual blocks, each containing two convolutional layers with 64 filters and a kernel size of 3. The dilation rates increased exponentially as 1, 2, 4, and 8. Each layer applied weight normalization and ReLU activation, with dropout (rate = 0.2) introduced to prevent overfitting. The baseline Transformer was configured with four encoder layers, each containing an 8-head self-attention mechanism and a feed-forward network (dimension 512), utilizing layer normalization and residual connections. Learnable positional encoding was applied, and classification was achieved through global average pooling and a linear layer. For hyperparameter tuning, we detail key configurations for each model while maintaining fairness. All models share a learning rate of 0.001 (0.0005 for Transformer with cosine annealing scheduling), a batch size of 64, and 100 training epochs; The random seed is uniformly set to 42 to ensure reproducibility. Note that RF uses its built-in iterative process and does not converge at a fixed number of iterations.

**Figure 7:**
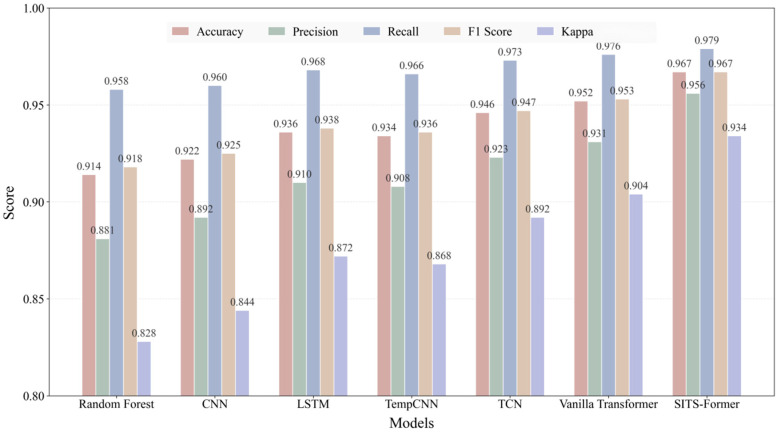
Performance comparison of different models.

The experimental results are shown in Figure 7. SITS-Former significantly outperforms the comparison baselines across all metrics, achieving an accuracy of 0.967. Notably, the model achieves a recall of 0.979, demonstrating its ability to effectively identify the vast majority of rice field areas while minimizing missed detections. This outstanding performance primarily stems from the model’s innovative spatio-temporal attention fusion mechanism. This design enables adaptive capture of critical spatio-temporal features throughout the rice growth cycle, demonstrating distinct advantages in handling long sequences, complex dynamics, and spatial dependencies. These experimental results fully validate SITS-Former’s technological advancement as a next-generation remote sensing time series analysis tool. Its exceptional performance provides a reliable technical foundation for crop monitoring in precision agriculture.

Based on pairwise comparisons of classification performance between the SITS-Former model and six baseline models (RF, CNN, LSTM, TempCNN, TCN, and Vanilla Transformer) on the same test set, this study employed the McNemar test to analyze the consistency of model predictions statistically (Table 4). This test evaluates whether performance differences between models are statistically significant by examining the number of samples where predictions diverge from those of the SITS-Former model.

The test results demonstrate that all comparisons achieved extremely high statistical significance (*p* < 0.0001), consistently confirming the superior performance of the SITS-Former model over all benchmark models. Specifically, in comparisons with models such as Random Forest, CNN, LSTM, and TempCNN, the results of inconsistent pairs strongly favored the SITS-Former model. That is, the number of pairs in which SITS-Former was correct and the baseline model was incorrect far exceeded the opposite scenario, as reflected in its exceptionally high chi-square value, indicating a substantial gap in performance between the two. However, when compared against more complex deep learning architectures like TCN and Vanilla Transformer, while statistical significance persists, the chi-squared values show a noticeable decline. This trend indicates that SITS-Former’s performance advantage narrows relative to these powerful benchmarks, suggesting that marginal gains in performance may begin to diminish once model architectures reach a certain level of complexity. Overall, the McNemar test not only establishes the statistical robustness of SITS-Former’s performance advantage but also reveals that its magnitude varies across different comparison models. This provides in-depth quantitative evidence for understanding the relative efficacy of this model.

### 4.3. Ablation Experiment

To objectively evaluate the independent contributions and synergistic effects of the proposed temporal features and model architecture in the task of identifying fragmented paddy fields, this section details two sets of systematic ablation experiments.

#### 4.3.1. Feature Combination Ablation Study

This study employs a systematic feature ablation experiment to empirically evaluate the contribution of introducing the two temporal-derivative features, TDVI and NCRVI. The experiment was conducted using a progressive feature combination strategy under the SITS-Former architecture (Table 5). The baseline model, which included only traditional vegetation indices and their temporal gradients, achieved an accuracy of 0.922, establishing a benchmark for subsequent improvements.

The first significant performance leap came with the introduction of TDVI (accuracy: 0.948), highlighting its superior capability in capturing abrupt spectral-moisture responses associated with key phenological events such as transplanting and heading. Subsequently, the separate introduction of NCRVI also yielded considerable gains (accuracy: 0.942), confirming its effectiveness in characterizing rice biomass accumulation processes by quantifying the continuous change dynamics throughout the entire growth cycle.

Ultimately, the integration of both TDVI and NCRVI enabled the model to achieve peak performance with an accuracy of 0.967. This optimal result reveals the inherent complementarity between the two features: TDVI excels at detecting transient phenological phase transitions, while NCRVI is adept at depicting progressive growth patterns across the growing season. This synergistic mechanism allows the model to leverage both localized abrupt signals and global trend information, thereby exhibiting enhanced robustness and higher classification accuracy when confronted with phenological variations induced by diverse environmental conditions.

#### 4.3.2. Model Architecture Ablation Study

The model architecture ablation study was conducted to dissect the necessity of each innovative component within the SITS-Former model. Experiments were performed by progressively introducing individual modules, starting from a baseline using the aforementioned complete feature set. The results (see Table 6) indicate that the original SITS-Former benchmark model achieved an accuracy of 0.895. Introducing the multi-scale embedding module resulted in the most significant performance leap, with accuracy substantially increasing to 0.932. This highlights the importance of capturing phenological patterns at different temporal scales. Subsequently, the incorporation of adaptive positional encoding led to a slight accuracy improvement to 0.908, demonstrating its effectiveness in modeling irregular time series data. Further integration of the phenological attention gating mechanism boosted accuracy to 0.913, with this module effectively focusing the model’s attention by emphasizing critical phenological stages. Finally, the complete SITS-Former model, integrating all improved components, achieved optimal performance across all evaluation metrics with an accuracy of 0.967, fully validating the comprehensiveness and sophistication of the proposed model architecture.

### 4.4. Performance Comparison and Advantage Analysis

**Figure 8:**
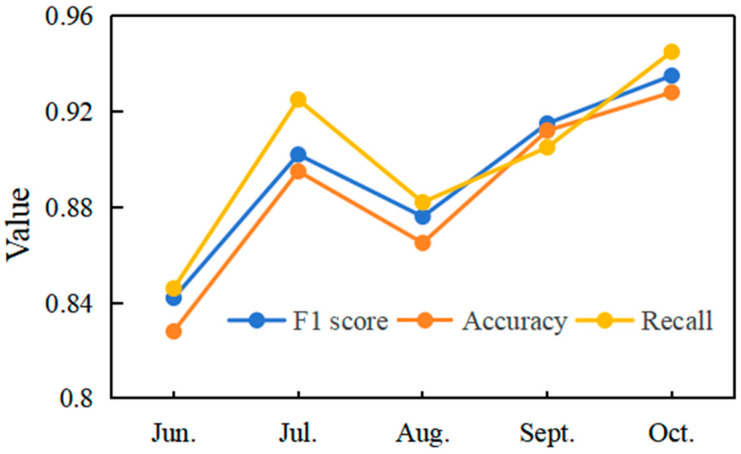
Comparison of Classification Performance Across Rice Growth Stages.

To verify whether the proposed method of building multi-vegetation index fusion temporal derivative features improves the accuracy of rice cultivation area identification, this study used the CNN classification model introduced in Section 4.2. When only the feature set built from a single growth stage of the multi-vegetation index was used, the detailed classification results and accuracy metrics are presented in Figure 8.

The results show that both accuracy and recall with the proposed time series method exceed methods that rely on a single growth stage to construct multi-vegetation index features.

In June, when the study area rice enters the early transplanting stage, the plants are short, and the canopy is not yet fully developed. At this stage, the NDVI of rice is typically low, around 0.3–0.4, indicating sparse vegetation cover. This spectral characteristic is highly similar to that of surrounding weeds, which often exhibit NDVI values between 0.25 and 0.35. This narrow spectral gap leads to severe overlap, making it difficult to distinguish rice from non-rice plots solely based on June imagery. The spectral and textural characteristics are not mature, causing substantial spectral confusion with surrounding weeds and other non-rice plots. Therefore, relying only on data from this stage yields an accuracy of 0.824, and accuracy remains low throughout the growth cycle.

By August, the rainy season brings more cloud cover and atmospheric scattering, which degrade the radiometric accuracy and spatial resolution of optical remote sensing data. Spectral information that was clearly discernible in some rice fields becomes obscured, increasing the difficulty of spectral unmixing and rice identification. At this point, using single-stage data for rice identification drops the accuracy to 0.875, lower than the accuracy in July, September, and October. Furthermore, even when clear data is available, the spectral reflectance in August, representing peak growth, might be affected by phenological delays caused by excessive rainfall. Such delays can alter the typical curve shape of vegetation indices, for example, shifting the peak reflectance or slowing down the senescence phase. This deviation from the expected spectral signature further complicates single-stage analysis, as the observed spectral information might not accurately reflect the “typical” rice spectrum for that month.

This study uses time series data to track the entire growth cycle—from transplanting through growth to maturity. It captures every small change in rice development at each stage, and the dataset shows how rice spectral features change as it grows. By combining details from different time points, this method solves two problems: single time point data can hide early growth features, and bad weather can degrade mid-growth data quality. This approach addresses both.

**Figure 9:**
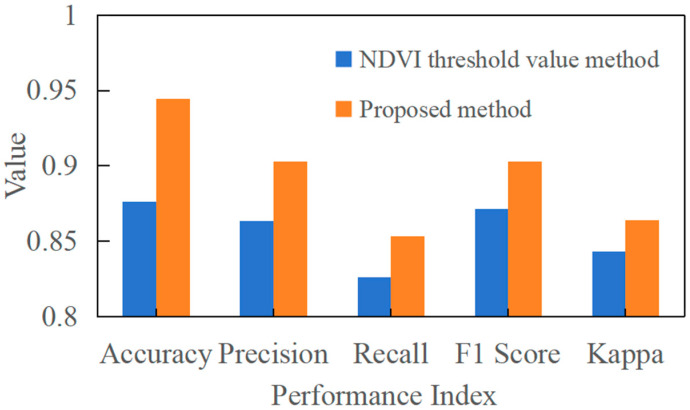
Comparison of Classification Results and Accuracy Metrics Between the Proposed Method and the Single Time Series NDVI Threshold Method.

We also compared results with those from using only NDVI thresholds. The specific classification results and accuracy numbers are shown in Figure 9.

The proposed method demonstrates superior performance over the single NDVI threshold approach, achieving significantly higher accuracy, recall, and F1-score. While temporal NDVI data can track rice growth patterns, methods that rely on a single threshold face inherent limitations because the spectral signatures of other vegetation types often resemble those of rice, thereby reducing identification accuracy. Our approach addresses this fundamental limitation through an improved architectural design that achieves a rice identification accuracy of 0.944. This enhanced performance stems from the method’s ability to eliminate spectral confusion while providing more robust data support for precise rice field mapping and large-area growth monitoring.

The discriminative power of our method derives from rice’s distinct phenological trajectory, which exhibits characteristic patterns throughout the growing season. Although rice and other mature crops might reach similar NDVI peak values during August, their complete seasonal patterns differ substantially in critical aspects, including the rate of vegetation index increase preceding the peak and the subsequent decline during senescence. The single NDVI threshold method cannot adequately capture these temporal nuances, particularly when other vegetation types spectrally mimic rice at certain growth stages. By integrating information across the complete phenological cycle, our time series fusion approach effectively captures these subtle temporal differences, thereby achieving superior accuracy and enhanced robustness against spectral confusion.

The Wuxi study area presents particular challenges for remote sensing monitoring due to characteristic summer conditions that include high temperatures, heavy precipitation, and extensive cloud cover. These climatic conditions frequently obscure crucial spectral signatures during August when rice reaches peak growth, while early-season data from June exhibit limited discriminative capacity owing to low plant density and spectral interference from other vegetation. Our methodology successfully overcomes these limitations by utilizing complete vegetation index time series that capture the full phenological progression of rice from germination through establishment, flowering, and senescence. This comprehensive temporal approach effectively addresses both the spectral interference present in early growth stages and the data degradation caused by adverse weather conditions during critical developmental periods.

### 4.5. Rice Mapping

**Figure 10:**
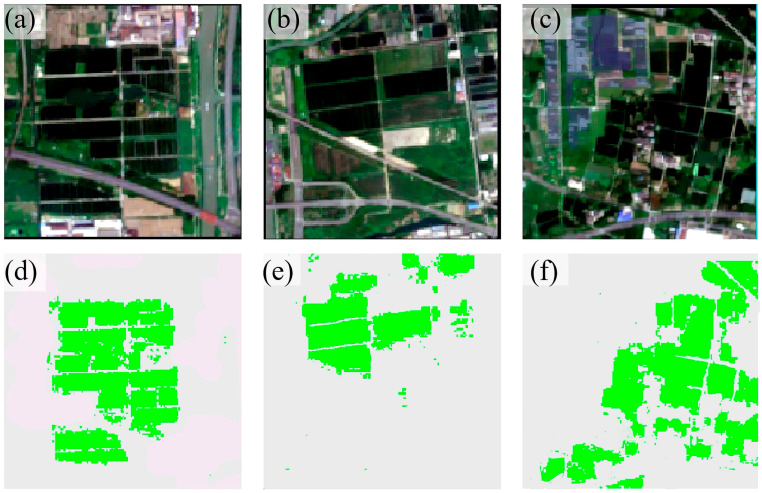
Spatial distribution maps of rice in different regions. (**a**–**c**) show Sentinel images; (**d**–**f**) present the results of rice identification.

Figure 10 displays the classification results of the model developed in this study for selected areas in Wuxi City. Rice fields in the Wuxi region exhibit highly fragmented distribution patterns with significant spatial heterogeneity, imposing stringent demands on the spatial detail recognition capability of remote sensing classification models. To systematically evaluate the model’s robustness in such complex scenarios, a regional cropping strategy based on land cover types was adopted during sample construction: the study area was first stratified by primary land categories, and three sub-regions within each stratum were randomly selected as evaluation samples. This approach ensures sample representativeness and land-type coverage while effectively mitigating selection bias. Experimental results demonstrate that despite practical challenges such as fragmented plots and non-contiguous distribution, the proposed model maintains high classification accuracy. It proves strong recognition of both the boundaries and internal structures of scattered rice fields, validating the model’s applicability and stability in complex cropping environments.

**Figure 11:**
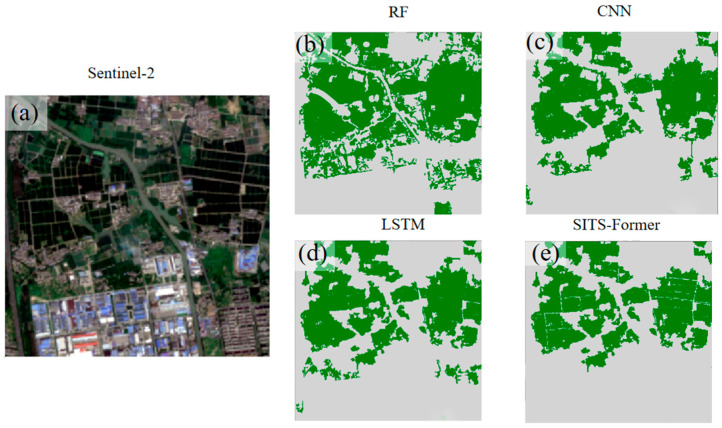
Rice Identification Effectiveness in Selected Areas of Wuxi City. (**a**) shows the Sentinel-2 impact on selected areas of Wuxi City in September 2023; (**b**–**e**) Classification results of different models in the study area.

To better illustrate the model’s rice classification ability, we selected specific areas within Wuxi City and cropped them to 270 × 270 pixels, resulting in 72,900 sample points. Then, we used four different methods to identify and extract the selected area (Figure 11).

As shown in Figure 11, our method demonstrates significantly superior performance compared to RF and other baseline methods. It has high recognition accuracy. But some dirt roads in the fields are narrow. Weeds cover these roads, mixing with rice plants on both sides. So some of these dirt roads are not recognized.

**Figure 12:**
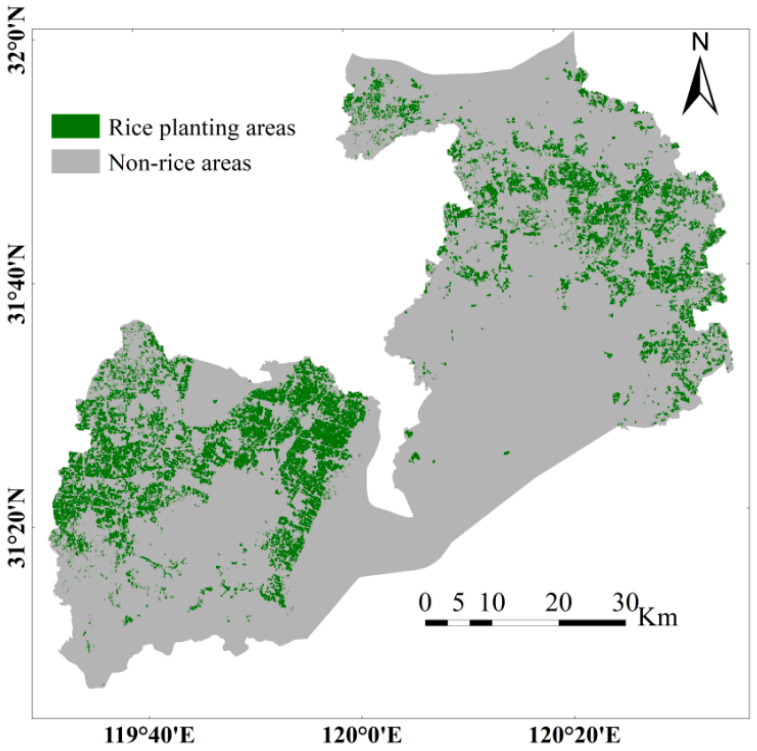
Distribution Map of Rice Cultivation in Wuxi City, 2023.

Based on an optimized model trained with extensive data, we produced a detailed map of the 2023 rice cultivation zones in Wuxi City. Post-processing removed small pixel clusters and scattered noise, with final identification results illustrated in Figure 12. This spatial distribution reveals rice fields mainly distributed across the western and northeastern zones. Accelerated urbanization has converted central areas for development, significantly reducing available farmland.

### 4.6. Reliability Verification and Analysis of Mapping Results

**Figure 13:**
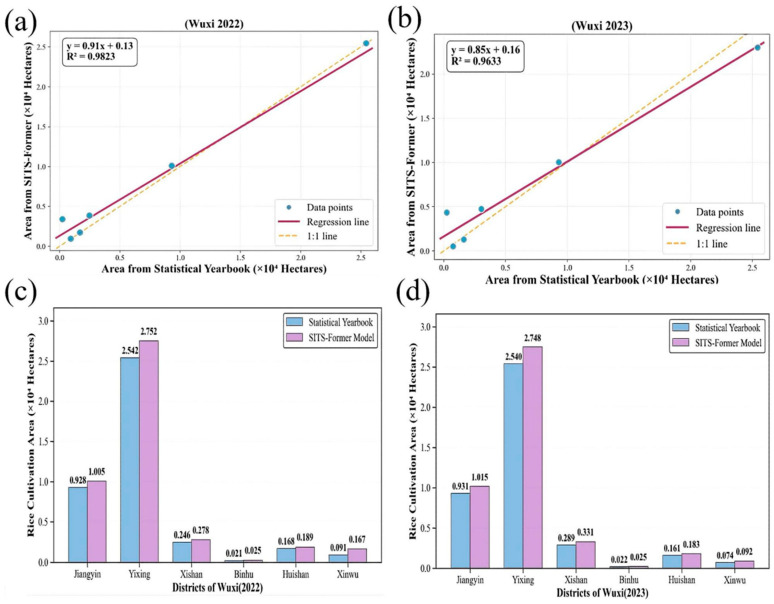
Validation and County-level Comparison of Rice Cultivation Area Estimated by SITS-Former Model in Wuxi (2022–2023): (**a**) Scatter Plot of SITS-Former-predicted vs. Statistical Yearbook Rice Area in Wuxi (2022); (**b**) Scatter Plot of SITS-Former-predicted vs. Statistical Yearbook Rice Area in Wuxi (2023); (**c**) County-level Rice Area Comparison between Statistical Yearbook and SITS-Former Model (Wuxi, 2022); (**d**) County-level Rice Area Comparison between Statistical Yearbook and SITS-Former Model (Wuxi, 2023).

The SITS-Former model proposed in this study demonstrates excellent performance in identifying fragmented paddy fields in the Wuxi area. To systematically evaluate its classification accuracy and generalization capability, we conducted a multi-dimensional comparison between its outputs and official statistical yearbook data (Figure 13).

As shown in Figure 13a,b, the model-estimated rice areas for each district in both 2022 and 2023 show strong agreement with statistical data, with coefficients of determination (R^2^) reaching 0.9823 and 0.9633, respectively. Although the regression slope for 2023 is slightly less than 1, indicating a minor systematic underestimation, the model maintains stable performance across both years, demonstrating good temporal generalization.

To quantitatively assess the spatial pattern of farmland fragmentation, this study employed a combined approach of high-resolution remote sensing imagery interpretation, vector mapping, field surveys, and statistical data analysis. Using 2023 data, we analyzed Xinwu District of Wuxi City as a representative case. The interpretation of high-resolution imagery yielded the following farmland patch statistics: approximately 183 patches were smaller than 0.3 ha, accounting for about 24.83% of all patches but covering only some 28 ha (roughly 3.8% of the total farmland area); around 466 patches ranged between 0.3 and 2 ha, representing about 63.23% of the patch count and approximately 443 ha (nearly 59.86%) of the area; and only about 64 patches exceeded 2 ha in size, comprising roughly 8.68% of patches yet encompassing around 155 ha (about 20.9%) of the total farmland area. Additionally, random sampling and visual interpretation were conducted in Xishan, Binhu, and Huishan Districts, with results similarly showing significantly smaller mean patch sizes in these urban districts, further reflecting the high degree of farmland fragmentation intensified by urban expansion. This distribution pattern, characterized by many small patches and few large ones, typifies the highly fragmented agricultural landscapes observed in regions such as Wuxi. Such fragmentation is a structural outcome influenced by historical land-contracting policies and ongoing urbanization.

The model also shows strong spatio-temporal generalization. Spatially, validation across six districts in Wuxi confirms the geographic adaptability of the model. For the major cultivation district of Yixing, yearbook data over two years are 2.542 and 2.540 × 10^4^ ha, while the model estimates are 2.752 and 2.748 × 10^4^ ha, indicating reliable accuracy in large-scale cultivation areas. Regions such as Binhu and Xinwu, which are smaller and highly fragmented, also demonstrate strong adaptability.

Based on model predictions, Wuxi’s 2023 rice acreage is estimated at 4.394 × 10^4^ ha. According to the 2024 Wuxi Statistical Yearbook, which reports official data of 4.031 × 10^4^ ha, the model’s recognition accuracy reaches 91.74%, underscoring its overall precision, particularly in capturing fragmented farmland.

## 5. Discussion

The SITS-Former model developed in this study demonstrates remarkable spatiotemporal robustness in identifying fragmented paddy fields in Wuxi, showing high consistency with official statistical yearbooks and demonstrating its potential for generalization in complex agricultural landscapes. However, current work still has room for improvement in model comparison, cross-crop applicability, sensor scalability, and multi-year validation. Compared to traditional machine learning methods (e.g., RF) and other temporal models (e.g., TempCNN, TCN, Vanilla Transformer), SITS-Former holds distinct advantages in overall accuracy and sensitivity to fragmentation, particularly excelling in capturing paddy fields within highly fragmented areas. However, local errors in certain areas remain influenced by fragmentation levels, terrain heterogeneity, and inter-annual variations in data quality, necessitating in-depth diagnostics through refined regional partition statistics and residual analysis. Such analyses hold significant implications for future method improvements.

Furthermore, while this study employs temporal vegetation indices such as TDVI and NCRVI to describe growth dynamics, Rana et al. (2024) suggest that extracting time-dependent crop growth signals directly from multi-date segmentation masks can also yield valuable temporal information [59]. This complements our existing spectral index-based temporal features and may offer a more intuitive spatiotemporal evolution perspective in certain scenarios. Therefore, it is recommended that future work incorporate temporal information from segmentation masks into models or use them as an alternative comparison method to more comprehensively characterize crop growth dynamics. Specifically, this could involve parallel integration of the temporal evolution features of segmentation masks at the data level, or adding a dedicated module within the model to directly account for mask time series. This approach would enhance robustness and interpretability under cloud cover and fragmented conditions.

Regarding the method’s general applicability, SITS-Former was designed for flexible integration of time series features and multi-source information. Thus, it is theoretically scalable to other crop identification and area estimation tasks, as well as adaptable to different sensor data (e.g., Sentinel-1 and Landsat). Future research should systematically evaluate the method’s transferability to staple crops such as wheat and maize across diverse ecological regions and assess its performance within multi-sensor fusion frameworks to determine whether and under what conditions it can replace or supplement existing crop monitoring systems. Simultaneously, extending applications to other high-cloud imagery environments requires in-depth analysis of how sensor band combinations, resolution, and temporal density impact performance.

Multi-year validation is crucial for enhancing application reliability. Current results are based on a cross-sectional comparison of two years’ data. Future work should conduct longitudinal validation using multi-year, multi-regional datasets to assess the model’s robustness and generalizability across factors such as variations in crop size, differences in sowing dates, cropping systems, and production regimes. For production planning and agricultural policy, establishing multi-year error distribution models and quantifying uncertainty would empower policymakers to conduct more reliable arable land resource management and yield forecasting under varying climate scenarios.

## 6. Conclusions

The SITS-Former model developed in this study employs self-attention mechanisms to analyze vegetation index time series, effectively identifying key phenological nodes through temporal correlations. This architecture demonstrates robust performance under limited training samples and class imbalance conditions, proving particularly effective in cloud-prone regions. We innovatively combined NDVI, LSWI, and EVI with temporal derivatives (TDVI and NCRVI) to optimize feature representation. This integration comprehensively captures rice growth dynamics through three-dimensional features describing growth rate, relative change, and spectral response, significantly improving detection sensitivity in fragmented farmlands and marginal cultivation zones.

Experimental results show our multi-index temporal fusion strategy outperforms single vegetation indices in classification accuracy. Compared to RF, CNN, LSTM, TCN and Vanilla Transformer models, SITS-Former achieved superior performance in accuracy (91.74%), F1 score, and recall when mapping rice areas in Wuxi (total detected area: 4.394 × 10^4^ ha). The model effectively minimizes misclassification in complex environments while maintaining operational efficiency. This methodology provides a practical framework for rice monitoring and precision agriculture management in Jiangsu Province and the Yangtze River Plain regions, with direct applications in yield prediction, irrigation planning, and land resource governance.

## Figures and Tables

**Table 1:** The remote sensing imagery data selected for this study.

Sensor	Bands	Spatial Resolution	Temporal Resolution
Sentinel-2 MSI	Coastal aerosol	60 m	5 d
	Blue	10 m	
	Green	10 m	
	Red	10 m	
	Red Edge_1	20 m	
	Red Edge_2	20 m	
	Red Edge_3	20 m	
	NIR_1	10 m	
	NIR_2	20 m	
	Water Vapor	60 m	
	SWIR_1	60 m	
	SWIR_2	20 m	
	SWIR_3	20 m	

**Table 2:** Details of the Selected Sentinel-2 MSI Imagery.

Sensor	Rice Phenophases	Time	Quantity
Sentinel-2 MSI	Transplantation period	June 2022	4
		June 2023	4
	Growth Period	July 2022	2
		August 2022	4
		September 2022	4
		July 2023	4
		August 2023	4
		September 2023	4
	Maturation period	October 2022	4
		October 2023	4

**Table 3:** Assessment indicators used in this study.

Metric	Formula
Accuracy	Accuracy = $\frac{TP+TN}{TP+TN+FP+FN}$
Precision	Precision =$\;\frac{TP}{TP+FP}$
Recall	Recall =$\;\frac{TP}{TP+FN}$
F1 Score	F1 Score =$\;\frac{2\;\times \;Precision\;\times \;Recall}{Precision\;+\;Recall}$
Kappa	Kappa =$\;\frac{p_{o}-p_{e}}{1+p_{e}}$ $p_{e}\;$=$\;\frac{\left(TP+FP)(TP+FN)(TN+FP)(TN+FN\right)}{{\left(TP+TN+FP+FN\right)}^{2}}$

**Table 4:** Results of the McNemar paired test between SITS-Former and comparison models on the test set.

Model	Error Count	Discordant Pair Count	SITS-Former Correct, Baseline Wrong	SITS-Former Wrong, Baseline Correct	χ^2^ Value	*p*-Value
Random Forest	516	378	348	30	280.13	<0.0001
CNN	468	340	305	35	213.98	<0.0001
LSTM	384	276	231	45	124.33	<0.0001
TempCNN	396	284	241	43	138.21	<0.0001
TCN	324	286	206	80	54.66	<0.0001
Vanilla Transformer	288	330	210	120	23.98	<0.0001

**Table 5:** Feature Combination Ablation Study Results.

	Accuracy	Precision	Recall	F1 Score	Kappa
Baseline Features	0.922	0.892	0.885	0.8601	0.8100
+TDVI	0.948	0.926	0.908	0.911	0.902
+NCRVI	0.942	0.906	0.899	0.902	0.894
Complete Feature Sat	0.967	0.956	0.979	0.967	0.934

**Table 6:** Model Architecture Ablation Study Results.

	Accuracy	Precision	Recall	F1 Score	Kappa
Original SITS-Former	0.895	0.872	0.885	0.870	0.842
+Multi-scale Embedding	0.932	0.941	0.937	0.939	0.921
+Adaptive Positional Encoding	0.908	0.893	0.890	0.891	0.865
+Phenological Attention Gate	0.913	0.898	0.899	0.898	0.871
Proposed Improved SITS-Former	0.967	0.956	0.979	0.967	0.934

## Data Availability

The data in this paper can be found at the following link: https://github.com/xutiantian0221/rice_model (accessed on 12 October 2025). Due to data protection, this data is currently private. You can contact the author to request access to the data if needed.
